# Metabolic syndrome‐related SNPs in HLA and TNF7L2 may be risk factors for generalized pustular psoriasis in Chinese Han population

**DOI:** 10.1002/ski2.18

**Published:** 2021-05-02

**Authors:** Y. Liu, Z.‐Y. Cui, J. Bao, X.‐L. Zhang, Y. Guo, M.‐J. Su, J.‐W. Han

**Affiliations:** ^1^ Department of Dermatology Affiliated Hospital of Inner Mongolia Medical University Hohhot Inner Mongolia China

## Abstract

**Background:**

Generalized pustular psoriasis (GPP) is a rare and severe type of psoriasis. Previous studies have reported that metabolic syndrome and its components have been associated with psoriasis.

**Objective:**

To investigate the association of metabolic syndrome‐related single‐nucleotide polymorphisms (SNPs) and GPP in Chinese Han population.

**Materials and Methods:**

One hundred and thirty‐six (136) GPP patients and 965 healthy controls were recruited in the study. Approximately, 4 ml peripheral venous blood was collected from each participant. After collection, second‐generation sequencing was used to detect genetic polymorphism of 15 SNPs. The plink 1.07 software package was used for statistical analysis.

**Results:**

Rs805303 (*p* = 0.01, OR = 0.70) and rs3177928 (*p* = 3.18E−07, OR = 2.66) in HLA were significantly different between the two groups. Moreover, rs4506565 (*p* = 1.41E−03, OR = 2.72) and rs7901695 (*p* = 9.39E−04, OR = 2.82) in TCF7L2 were significantly associated with GPP in patients without a previous history of PsV. Genotype analysis of rs4506565 and rs7901695 showed that under the recessive model, genotype frequencies of rs4506565 (*p* = 0.00, OR = 18.52) and rs7901695 (*p* = 0.00, OR = 18.44) were significantly different between GPP patients and healthy controls.

**Conclusion:**

Rs805303 and rs3177928 in HLA may increase the risk of GPP in the Chinese Han population. TCF7L2 may be a risk factor for GPP in patients without a previous history of PsV.

## Graphical abstract

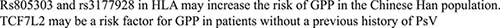

## 1 INTRODUCTION

Psoriasis is a common and complex inflammatory disease. Four clinical types of psoriasis include psoriasis vulgaris, psoriatic arthropathy, psoriatic erythroderma and pustular psoriasis. Generalized pustular psoriasis (GPP) is a recognized subtype of pustular psoriasis (PP); however, this severe type of psoriasis is very rare.

**What is already known about this topic?**

- Metabolic syndrome and its components (dyslipidaemia, hypertension, insulin resistance, abdominal obesity) are associated with the occurrence of psoriasis.
- GPP is a rare type of psoriasis; the genetic mutations of GPP are different compared to psoriasis vulgaris.

**What does this study add?**

- In the Chinese Han population, rs805303, rs3177928 in HLA may be risk factors for GPP, while TCF7L2 may be a risk factor for GPP without a history of PsV.
- Under the recessive model, two SNPs in TCF7L2 could increase the risk of GPP.

Various metabolic diseases, such as obesity, diabetes and cardiovascular disease factors, have been associated with psoriasis.^1^ The metabolic syndrome, which is a multiplex risk factor for atherosclerotic cardiovascular disease and Type 2 diabetes, contains a clustering of abnormalities such as insulin resistance, dyslipidaemia, hypertension and central obesity.^2^ Some researchers have reported that metabolic syndrome, as well as its components, are associated with psoriasis.^3^ Besides, shared susceptible genetic variants have been reported in some studies. For example, a meta‐analysis has summarized the top 10 single‐nucleotide polymorphisms (SNPs) of metabolic disease associated with psoriasis.^4^ Moreover, Cheng et al.^5^ found an association between Rs2303138 in the LNPEP gene, which has a role in hypertension and diabetes, and psoriasis. However, so far, rare studies have reported on the association between metabolic syndrome components and GPP.

The pathogenesis of GPP still remains somewhat unclear. Previous studies have suggested that GPP can present in patients with or prior history of psoriasis vulgaris (GPP with PsV), but also in those without a history of psoriasis vulgaris (GPP without PsV). Besides, studies indicated that the genetic characteristic of GPP alone and GPP with PsV are different.^6^ The aim of the present study was to investigate the susceptible genes of metabolic syndrome components in Chinese Han patients with GPP.

## 2 MATERIALS AND METHODS

### 2.1 Subjects

A total of 136 GPP patients and 965 healthy controls were enrolled in this retrospective study. All the GPP patients were hospitalized with body surface area (BSA) more than 50% from December 2012 to March 2019. Patients' demographic and clinical characteristics such as onset of age, family history, were interviewed (Table 1). Healthy controls were selected from the physical examination centre in the same period. Inclusion criteria were as follows: (1) the participants were between 18 and 80 years of age; (2) GPP confirmed by clinical (at least two dermatologists) and pathohistological examination; (3) patients who signed the informed consent. All of the healthy controls recruited in this study were individuals without psoriasis, without autoimmune disorders or systemic disorders and no family history of psoriasis (including first‐, second‐ and third‐degree relatives).

**TABLE 1:** Demographic and clinical characteristics of GPP patients

Characteristics	Total GPP	GPP without PsV	GPP with PsV
Numbers of patients (male/female)	136 (65/71)	58 (23/35)	78 (42/37)
Family history	No	No	No
Mean age	45.799 ± 15.003	46.526 ± 13.489	45.747 ± 13.554
Mean onset of GPP	32.993 ± 16.037	37.281 ± 16.874	29.859 ± 14.728

Exclusion criteria: individuals with other forms of psoriasis (plaque, droplet, erythrodermic, inverse and arthritic) or other autoimmune diseases; those who received any systemic therapy (corticosteroids, immunosuppressive medications, retinoids, methotrexate, biological therapy) for 4 weeks before study participation. Individuals (patients and controls) who have metabolic disorders (such as hyperglycaemia, dyslipidaemia, hypertension) as well as those individuals with primary hypertension, Type 1 diabetes, familial hyperlipidaemia were excluded in our study.

The study was approved by the Ethical Committee of the Affiliated Hospital of Inner Mongolia medical university and was conducted according to Declaration of Helsinki principles.

### 2.2 Blood collection and analysis

Approximately 4 ml of peripheral venous blood was collected from each participant. After collection, samples were transferred into tubes containing Edtap dipotassium ethylene diamine tetraacetate (K_2_EDTA) and then stored at −80 °C for subsequent DNA extraction.

### 2.3 Selection of SNPs

Based on previous studies, 15 SNPs in different gene regions were selected, which were also reported associated with psoriasis. Including the top 10 SNPs associated with psoriasis summarized by Lu et al.^4^ rs5186 in AT1R by Mohammadi et al.^7^ rs2303138 in the LNPEP gene by Cheng et al.^5^ (Table 2). All these genes were related with metabolic syndrome or its components (such as hyperglycaemia, dyslipidaemia and hypertension) in genetics or function.

**TABLE 2:** Fifteen SNPs selected through literature search

CHR	SNP	BP	Gene	Literature
2	rs7593730	160314943	*RBMS1*	Lu et al.^4^
3	rs5186	148742201	*AT1R*	Mohammadi et al.^7^
5	rs2303138	97015006	*LNPEP*	Cheng et al.^5^
6	rs805303	31616366	*HLA*	Lu et al.^4^
6	rs3177928	32412435	*HLA*	Lu et al.^4^
6	rs2247056	31265490	*HLA*	Lu et al.^4^
10	rs4506565	112996282	*TCF7L2*	Lu et al.^4^
10	rs7901695	112994329	*TCF7L2*	Lu et al.^4^
12	rs3184504	111446804	*SH2B3*	Lu et al.^4^
12	rs653178	111569952	*ATXN2, SH2B3*	Lu et al.^4^
12	rs11065987	111634620	*BRAP*	Lu et al.^4^
16	rs255049	67979568	*LCAT*	Lu et al.^4^
17	rs4646994	63488539 : 63488540	*ACE*	Simsek et al.^8^
19	rs492602	48703160	*FUT2*	Hoffmann et al.^9^
22	rs181362	21577779	*UBE2L3*	Lu et al.^4^

Abbreviations: BP, biological position; CHR, chromosome; SNP, single‐nucleotide polymorphism.

Genomic DNA was extracted using a whole blood DNA extraction kit (AxyPrep, AP‐MX‐BL‐GDNA‐25). The primers and probes were designed using Primer3 online (version 0.4.0; http://frodo.wi.mit.edu/) and Oligo (version 6.31; Molecular Biology Insights, Inc. (DBA Oligo), respectively.

The polymerase chain reaction (PCR) system included: 1 μl of DNA template, 2 μl of the buffer, 0.6 μl Mg^2+^, 2 μl dNTP, 0.2 μl Taq DNA polymerase, 2 μl primer solution and 12.2 μl water (total volume was 20 μl). PCR condition was performed by initial denaturation at 95°C for 2 min, denaturation at 94°C for 30 s and annealing at 59°C for 90 s, 40 extension cycles at 72°C for 60 s, followed by another extension at 72°C for 10 min. Amplified products were simultaneously sequenced using next‐generation sequencing technology (PRISM3730 ABI). The PCR products were analysed by 0.8% agarose gel for assay integrity—preliminary analysis of sequencing results using the Ion Torrent PGM platform with its own analysis software. The DNA purification and sequencing were completed by the Shanghai YiHe application of Biotechnology.

### 2.4 Statistical analysis

The variants of SNPs were performed by Minor allele frequencies (MAF). MAF of the 15 SNPs was compared between the two groups. The genotype frequencies of 2 SNPs in transcription factor 7‐like 2 (TCF7L2) loci were calculated. Statistical software Plink1.07 (http://pngu.mgh.harvard.edu/purcell/plink/) was used to analyse the data in this study. A *χ* ^2^ test was performed to determine the MAF and genotype frequencies of each group, as well as odds ratio (OR) and its 95% confidence interval (95% CI). A *p* value of less than 0.05 was considered statistically significant.

## 3 RESULTS

Sixty‐five females and 71 males were recruited in the patients' group (mean age 45.799 ± 15.003 years), and 423 females and 542 males in the control group (mean age 45.865 ± 11.588 years). There was no difference in age and gender between the two groups (*p* > 0.05).

Two SNPs located in human leucocyte antigen (HLA) loci, that is, rs805303 (*p* = 0.01, OR = 0.70), rs3177928 (*p* = 3.18E−07, OR = 2.66) showed an obvious difference between groups; while no difference was found for others SNPs (*p* > 0.05; Table 3). The frequency of the rs3177928 A allele was significantly higher in the GPP patients (14.4%) compared with the control subjects (6.0%). On the other hand, the frequency of the rs805303 T allele was significantly lower in the GPP patients (34.4%) compared with that in controls (42.7%; Table 3).

**TABLE 3:** Comparison of metabolic associated risk SNPs with GPP and healthy controls

CHR	SNP	BP	Gene	Allele	MAF		*χ* ^2^	*p*	OR	95% CI
					Case‐control					
2	rs7593730	160314943	RBMS1	T	0.182	0.184	0.010	0.92	0.98	0.71–1.37
3	rs5186	148742201	AT1R	C	0.077	0.061	1.041	0.31	1.29	0.79–2.08
5	rs2303138	97015006	LNPEP	A	0.430	0.414	0.262	0.61	1.07	0.83–1.38
6	rs805303	31616366	HLA	T	0.344	0.427	6.578	0.01	0.70	0.54–0.92
6	rs3177928	32412435	HLA	A	0.144	0.060	26.140	3.18E–07	2.66	1.81–3.92
6	rs2247056	31265490	HLA	T	0.077	0.112	3.008	0.08	0.66	0.42–1.06
10	rs4506565	112996282	TCF7L2	T	0.059	0.041	1.800	0.18	1.46	0.84–2.53
10	rs7901695	112994329	TCF7L2	C	0.059	0.040	2.147	0.14	1.51	0.87–2.63
12	rs3184504	111446804	SH2B3	T	0.015	0.009	0.878	0.35	1.68	0.56–5.03
12	rs653178	111569952	ATXN2	G	0.015	0.008	1.145	0.29	1.81	0.60–5.46
12	RS11065987	111634620	BRAP	G	0.015	0.008	1.425	0.23	1.94	0.64–5.90
16	rs255049	67979568	LCAT	C	0.147	0.142	0.0445	0.83	1.04	0.73–1.49
17	rs4646994	63488539 : 63488540	ACE	G	0.283	0.269	0.208	0.65	1.08	0.79–1.46
19	rs492602	48703160	FUT2	C	0.007	0.018	1.562	0.21	0.41	0.10–1.73
22	rs181362	21577779	UBE2L3	A	0.455	0.422	1.040	0.31	1.14	0.88–1.48

Abbreviations: 95% CI, 95% confidence interval; BP, biological position; CHR, chromosome; MAF, minor allele frequency; OR, odds ratio; SNP, single‐nucleotide polymorphism.

After GPP patients were categorized into two groups, there were 78 GPP patients with existing or prior psoriasis vulgaris (GPP with PsV) and 58 GPP patients without a history of psoriasis vulgaris (GPP without PsV). Two SNPs in TCF7L2, that is, rs4506565 (*p* = 1.41E−03, OR = 2.72), rs7901695 (*p* = 9.39E−04, OR = 2.82) were significantly different between GPP patients without PsV and controls. Moreover, rs805303 in HLA was obviously different between GPP without PsV group and healthy controls. In contrast, rs3177928 in HLA was significantly associated with GPP with PsV (Table 4).

**TABLE 4:** Comparison of the 15 SNPs in GPP patients without PsV and those with PsV, with healthy controls

CHR	SNP	Gene	Allele	GPP without PsV		*χ* ^2^	p*	OR	95% CI	GPP with PsV		*χ* ^2^	p*	OR	95% CI
				Case	Control					Case	Control				
2	rs7593730	RBMS1	T	0.193	0.184	0.058	0.81	1.06	0.66–1.71	0.173	0.184	0.114	0.74	0.93	0.60–1.43
3	rs5186	AT1R	C	0.078	0.061	0.508	0.48	1.29	0.64–2.62	0.077	0.061	0.615	0.43	1.28	0.69–2.37
5	rs2303138	LNPEP	A	0.440	0.414	0.301	0.58	1.11	0.76–1.62	0.423	0.414	0.051	0.82	1.04	0.75–1.45
6	rs805303	HLA	T	0.325	0.427	4.615	0.03	0.65	0.43–0.97	0.358	0.427	2.660	0.10	0.75	0.53–1.06
6	rs3177928	HLA	A	0.105	0.060	3.840	0.05	1.86	0.99–3.47	0.173	0.060	29.240	6.38E–08	3.30	2.09–5.20
6	rs2247056	HLA	T	0.086	0.112	0.742	0.39	0.75	0.39–1.45	0.071	0.112	2.563	0.11	0.60	0.32–1.13
10	rs4506565	TCF7L2	T	0.105	0.041	10.200	1.41E‐03	2.72	1.44–5.15	0.026	0.041	0.934	0.33	0.61	0.22–16.8
10	rs7901695	TCF7L2	C	0.105	0.040	10.940	9.39E‐04	2.82	1.49–5.35	0.026	0.040	0.801	0.37	0.63	0.23–1.75
12	rs3184504	SH2B3	T	0.017	0.009	0.846	0.36	1.97	0.45–8.65	0.013	0.009	0.257	0.61	1.46	0.33–6.38
12	rs653178	ATXN2	G	0.018	0.008	1.053	0.30	2.13	0.48–9.40	0.013	0.008	0.366	0.55	1.57	0.36–6.90
12	RS11065987	BRAP	G	0.018	0.008	1.236	0.27	2.27	0.51–10.06	0.013	0.008	0.499	0.48	1.70	0.38–7.49
16	rs255049	LCAT	C	0.155	0.142	0.149	0.70	1.11	0.66–1.86	0.141	0.142	0.002	0.97	0.99	0.62–1.58
17	rs4646994	ACE	G	0.306	0.269	0.652	0.42	1.20	0.77–1.87	0.266	0.269	0.006	0.94	0.98	0.66–1.48
19	rs492602	FUT2	C	0.000	0.018	2.078	0.15	NA	NA	0.013	0.018	0.196	0.66	0.72	0.17–3.04
22	rs181362	UBE2L3	A	0.491	0.422	2.089	0.15	1.32	0.90–1.93	0.429	0.422	0.023	0	1.03	0.74–1.43

*Note*: *p < 0.05.

The genotypic association between the two SNPs in TCF7L2 and GPP risk was investigated in dominant and recessive models. The results showed that under the recessive model, genotype frequencies of the rs4506565 (TT) and rs7901695 (CC) were significantly higher in GPP patients than healthy controls (*p* = 0.00, OR = 18.52; *p* = 0.00, OR = 18.44, respectively), and especially in GPP patients without PsV (*p* = 0.00, OR = 46.30; *p* = 0.00, OR = 46.11, respectively). Moreover, trend frequencies of rs4506565 and rs7901695 were significantly associated with GPP without PsV in the trend model (*p* = 0.00, respectively; Table 5).

**TABLE 5:** Genotype analysis of rs4506565 and rs7901695 in TCF7L2

SNP	Genetic model	Genotype distribution		*χ* ^2^	*p*	OR	95% CI
		Case	Control				
GPP versus Control							
rs4506565	GENO (TT/TA/AA)	5/6/124	2/76/887				
	TREND	16/254	80/1850	1.626	0.20		
	DOM (TT + TA/AA)	11/124	78/887	0.001	0.98	1.01	0.52–1.95
	REC (TT/TA + AA)	5/130	2/963	22.989	0.00	18.52	3.56–96.43
rs7901695	GENO (CC/CT/TT)	5/6/124	2/73/886				
	TREND	16/254	77/1845	1.930	0.16		
	DOM (CC + CT/TT)	11/124	75/886	0.019	0.89	1.05	0.54–2.03
	REC (CC/CT + TT)	5/130	2/959	22.792	0.00	18.44	3.54–96.03
GPP without PsV versus Control							
rs4506565	GENO (TT/TA/AA)	5/2/50	2/76/887				
	TREND	12/102	80/1850	9.168	0.00		
	DOM (TT + TA/AA)	7/50	78/887	1.244	0.27	1.59	0.70–3.63
	REC (TT/TA + AA)	5/52	2/963	58.038	0.00	46.30	8.77–244.32
rs7901695	GENO (CC/CT/TT)	5/2/50	2/73/886				
	TREND	12/102	77/1845	9.782	0.00		
	DOM (CC + CT/TT)	7/50	75/886	1.456	0.23	1.65	0.73–3.78
	REC (CC/CT + TT)	5/52	2/959	57.787	0.00	46.11	8.74–243.31
GPP with PsV versus Control							
rs4506565	GENO (TT/TA/AA)	0/4/74	2/76/887				
	TREND	4/152	80/1850	0.926	0.34		
	DOM (TT + TA/AA)	4/74	78/887	0.031	0.86	1.08	0.48–2.42
	REC (TT/TA+AA)	0/78	2/963	0.162	0.69	1.00	1.00–1.01
rs7901695	GENO (CC/CT/TT)	0/4/74	2/73/886				
	TREND	4/152	77/1845	0.793	0.37		
	DOM (CC + CT/TT)	4/74	75/886	0.735	0.39	0.64	0.23–1.80
	REC (CC/CT + TT)	0/78	2/959	0.163	0.69	1.00	1.00–1.01

Abbreviations: GENO, genotype; DOM, dominant; REC, recessive.

## 4 DISCUSSION

Previous studies have suggested that GPP can occur with patients regardless of their history of PsV. Several factors, such as infections, drugs, psychological stress, hypocalcaemia and pregnancy, can trigger GPP. In addition, the three most common mutations related to GPP are located in IL36RN (encodes IL‐36 receptor antagonist), CARD14 (encodes a keratinocyte adaptor protein) and AP1S3 (encodes a subunit of the adaptor protein 1 complex). These three mutations account for less than 30% of GPP cases,^10^ which is different from that observed in patients with psoriasis vulgaris.

Inflammatory polyarthritis and metabolic syndrome are frequently associated with GPP. A previous study reported that obesity (43%), hypertension (26%), dyslipidaemia (26%) and diabetes (24%) are associated with GPP.^11^ In our study, two metabolic syndrome‐related SNPs (Rs805303 and rs3177928) in the HLA region resulted as risk factors for GPP. In the study of Lu et al.^4^ showed that rs3177928 is related to lipid metabolic, while rs805303 is related to blood pressure. HLA is already known to be a susceptibility loci in patients with psoriasis.^12^ HLA Class I and II alleles have been shown to be associated with GPP in the Japanese population.^13^ Nevertheless, the SNPs investigated in our present study have not yet been reported. We found that rs805303 is associated with GPP in patients without a previous history of PsV. As HLA is highly associated with psoriasis, disequilibrium reaction with other SNPs cannot be ruled out. So the specific relationship between HLA and GPP was uncertain, and further studies of different ethnicities are needed to establish whether this can be applied to global pollution in general.

Studies on various ethnic populations have confirmed that TCF7L2 SNPs are strongly associated with Type 2 diabetes (T2DM) risk.^14^ The variants of TCF7L2 have been related to impaired insulin secretion, increased hepatic glucose release during fasting, defects in incretin‐and glucose‐induced glucagon suppression. TCF7L2 can also affect insulin sensitivity and β‐cell development.^15^ Previous studies have suggested that the TCF7L2 gene variant is associated with psoriasis.^4^ Yet, a mutation in the TCF7L2 gene has been only marginally analysed in GPP. Rs4506565 and rs7901695 are located in the intron region of TCF7L2, the two SNPs has been much concerned by previous studies about the susceptibility with T2DM.^16^, ^17^ What is more, they were found in linkage disequilibrium with rs7903146 in some study,^15^ and rs7903146 was found significantly associated with T2DM in various ethnicities.^18^ In the present study, the two SNPs in TCF7L2 had a significantly higher prevalence in GPP patients without a previous history of PsV. As improper use of corticosteroids can induce the occurrence of GPP or diabetes, the relationship between TCF7L2 variants and GPP may occur due to glucose metabolism. Nevertheless, GPP does not frequently occur after inappropriate corticosteroid therapy in patients without a previous history of PsV. In our study, none of the participants have been using corticosteroids for at least 4 weeks. The results of the present study suggest that the variants of TCF7L2 are associated with the susceptibility of GPP. Moreover, genotype analysis demonstrated that rs4506565 and rs7901695 were associated with increased GPP risk in recessive models. The influence was much more obvious in GPP patients without a previous history of PsV.

This study has a few limitations. First, the sample size was not big enough. Second, this study included only Chinese Han patients.

In conclusion, our study showed that rs805303 and rs3177928 in HLA might increase the risk of GPP in the Chinese Han population. Besides, TCF7L2 may be a risk factor for GPP in patients without a previous history of PsV. Also, a recessive model may increase the risk of GPP. Yet, in order to identify the genetic association of TCF7L2 variants and GPP, further in‐depth studies are warranted in the future.

## CONFLICT OF INTERESTS

The authors declare that there are no conflict of interests.
